# Identification of a druggable protein–protein interaction site between mutant p53 and its stabilizing chaperone DNAJA1

**DOI:** 10.1074/jbc.RA120.014749

**Published:** 2020-11-21

**Authors:** Xin Tong, Dandan Xu, Rama K. Mishra, Ryan D. Jones, Leyu Sun, Gary E. Schiltz, Jie Liao, Guang-Yu Yang

**Affiliations:** 1 Department of Pathology, Northwestern University Feinberg School of Medicine, Chicago, Illinois, USA; 2 Robert H. Lurie Comprehensive Cancer Center, Northwestern University Feinberg School of Medicine, Chicago, Illinois, USA; 3 Center for Molecular Innovation and Drug Discovery (CMIDD), Northwestern University Feinberg School of Medicine, Chicago, Illinois, USA; 4 Department of Pharmacology, Northwestern University Feinberg School of Medicine, Chicago, Illinois, USA; 5 Department of Biochemistry and Molecular Genetics, Northwestern University Feinberg School of Medicine, Chicago, Illinois, USA

**Keywords:** DNAJA1, mutant p53, homology model, protein–protein docking, interacting pocket, pancreatic cancer, *in silico*, CHIP, C terminus of Hsc70-interacting protein, DMSO, dimethyl sulfoxide, DNAJA1, DnaJ homolog subfamily A member 1, FBS, fetal bovine serum, Hsp, heat shock protein, KO, knockout, mutp53, mutant p53, WT, wildtype, wtp53, wildtype p53

## Abstract

The *TP53* gene is the most frequently mutated gene in human cancers, and the majority of *TP53* mutations are missense mutations. As a result, these mutant p53 (mutp53) either directly lose wildtype p53 (wtp53) tumor suppressor function or exhibit a dominant negative effect over wtp53. In addition, some mutp53 have acquired new oncogenic function (gain of function). Therefore, targeting mutp53 for its degradation may serve as a promising strategy for cancer prevention and therapy. Based on our previous finding that farnesylated DNAJA1 is a crucial chaperone in maintaining mutp53 stabilization, and by using an *in silico* approach, we built 3D homology models of human DNAJA1 and mutp53^R175H^ proteins, identified the interacting pocket in the DNAJA1–mutp53^R175H^ complex, and found one critical druggable small molecule binding site in the DNAJA1 glycine/phenylalanine-rich region. We confirmed that the interacting pocket in the DNAJA1–mutp53^R175H^ complex was crucial for stabilizing mutp53^R175H^ using a site-directed mutagenesis approach. We further screened a drug-like library to identify a promising small molecule hit (GY1-22) against the interacting pocket in the DNAJA1–mutp53^R175H^ complex. The GY1-22 compound displayed an effective activity against the DNAJA1–mutp53^R175H^ complex. Treatment with GY1-22 significantly reduced mutp53 protein levels, enhanced Waf1p21 expression, suppressed cyclin D1 expression, and inhibited mutp53-driven pancreatic cancer growth both *in vitro* and *in vivo*. Together, our results indicate that the interacting pocket in the DNAJA1–mutp53^R175H^ complex is critical for mutp53’s stability and oncogenic function, and DNAJA1 is a robust therapeutic target for developing the efficient small molecule inhibitors against oncogenic mutp53.

The *TP53* gene is the most frequently mutated gene in human cancers; approximately 50% of human cancers have *TP53* alterations (1, 2). *TP53* encodes the p53 protein, which is a sequence-specific DNA-binding protein that regulates transcription of a number of downstream target genes involved in apoptosis, cell cycle arrest, and metabolism (3). The N terminus of p53 protein contains two transactivation domains, followed by a proline-rich domain, a highly conserved DNA-binding domain, and a C terminus encoding its nuclear localization signals and an oligomerization domain needed for transcriptional activity (4). The majority of *TP53* mutations are missense mutations in the DNA-binding domain, and among them, eight mutations (V157, R175, Y220, G245, R248, R249, R273, and R282) account for ∼28% of total mutations in *TP53* (5). There are two classes of p53 DNA-binding domain mutants, namely, conformational mutants and contact site mutants. The conformational mutants, for example, R175H and R249S, change the structure of p53 protein (6), whereas the contact site mutants, such as R248W and R273H, have an altered residue at a site that directly contacts DNA in the wildtype p53 (wtp53) protein (6, 7).

As a result, *TP53* missense mutation produces a full-length protein that can no longer bind DNA and is therefore incapable of transactivating its target genes, abrogating tumor suppressor activity of wtp53 (known as loss of function) (8, 9). Mutant p53 (mutp53) also exhibits a dominant negative effect through formation of a tetramer with wtp53, inhibiting the function of wtp53 (8, 9, 10, 11). Although carcinogenesis usually requires the loss of both alleles of most tumor suppressor genes, mutation of one allele of p53 can promote carcinogenesis owing to the dominant negative effect (12). Furthermore, some mutp53 may have gain-of-function properties, by which mutp53 acquires oncogenic function and promotes tumorigenesis, survival, invasion, and metastasis (13, 14, 15, 16). These mutp53 proteins accumulate in the cells and promote malignant progression. There is a crucial need to develop targeting therapy for mutp53. However, given the vast spectrum of mutations, therapies directly targeting mutp53 are extremely difficult and are unlikely to provide broad clinical utility (5, 17).

Unlike with wtp53, which is degraded shortly under unstressed conditions, these missense mutations can increase the stability of mutp53 protein (18). For example, mutp53 often loses its activity to interact with Mdm2 for degradation (19, 20). Consequently, these mutant versions of the p53 protein are commonly expressed at a high level in tumor. Protein folding and homeostasis are critically dependent on a complex network of molecular chaperones (21). Molecular chaperones generally make “triage” decisions regarding whether substrate proteins will be folded or degraded (22, 23), particularly for mutant proteins such as p53 (24, 25, 26). DNAJA1 (DnaJ homolog subfamily A member 1) is a crucial co-chaperone of heat shock protein 70 (Hsp70) (27, 28) and has been suggested to be a vital player for mutp53 stability and oncogenic function (24, 29). All the members of the DnaJ family contain the J domain, which primarily binds to Hsp70 and stimulates its ATPase activity (28). Besides the J domain, DNAJA1contains three other conserved regions including the glycine/phenylalanine rich domain, zinc finger domain, and C terminus (30). The C terminus possesses a CAAX motif, in which A is an aliphatic amino acid and X is any amino acid (31). This CAAX motif specifies the addition of a farnesyl group to the C (cysteine) of the CAAX motif, which allows binding to the client proteins and guidance of protein translocation (32). DNAJA1 is primarily a cytosolic protein, but the farnesylation allows it to be anchored to the endoplasmic reticulum and mitochondrial membranes (33). It has been reported that the mevalonate pathway is involved in mutp53 stabilization through inhibiting its ubiquitination by the CHIP (C terminus of Hsc70-interacting protein) E3 ubiquitin ligase in a manner relying on DNAJA1 (24). We further demonstrated that farnesylation of DNAJA1 CAAX motif is critical for its ability to stabilize mutp53, and inhibition of DNAJA1 farnesylation promotes mutp53 degradation and inhibits mutp53-driven carcinogenesis (29).

In the present study, by using an *in silico* approach, we built homology models and characterized druggable docking sites and interacting pockets of DNAJA1, mutp53^R175H^, and the DNAJA1–mutp53^R175H^ complex and verified their critical role in regulation of mutp53^R175H^ stabilization. Furthermore, we identified a promising small molecule hit (GY1-22) after screening a drug-like library against DNAJA1-mutP53^R175H^ interacting pocket, which can inhibit mutp53-driven pancreatic cancer cell growth both *in vitro* and *in vivo*.

## Results

### Building the homology models, identifying druggable binding sites and interacting pockets of DNAJA1, mutp53^R175H^, and the DNAJA1–mutp53^R175H^ complex by using an *in silico* approach

A previous report has indicated that DNAJA1, a molecular chaperone that belongs to the DnaJ/Hsp40 protein family, plays an important role in mutp53 stability by preventing ubiquitin-dependent mutp53 degradation (24). More recently, we have further demonstrated that the DNAJA1 C-terminal CAAX motif is critical for mutp53 degradation and inhibits mutp53-driven carcinogenesis in mice (29). Therefore, DNAJA1 and its mutp53 chaperone complex provide ideal druggable targets for eliminating oncogenic mutp53. In the absence of a crystal structure for DNAJA1, we built a homology model of the protein structure by considering the primary sequence of human DNAJA1 (NP_001530.1, 397 residues). The first step in the comparative homology model building method is to find the template structures of other proteins for which 3D structures have been solved. To this end, we carried out a BLAST/PSI-BLAST search, but this yielded no single template with sequence similarity >60% with DNAJA1. Hence, we used the multitemplate-based algorithm to build the 3D model of DNAJA1. Using the Prime module implemented in the Schrödinger platform (34), we built a comparative homology model, which was validated using MolProbity guidelines (35). Our MolProbity score was found to be >98th percentile (score >90th percentile indicates the model is of high quality and suitable for further *in silico* studies), as shown in Figure S1*A*. For mutp53^R175H^, the crystal structure was also not available; instead, a recent mutp53^R280K^ structure (6FF9.pdb) was available. Thus, we switched back the K280 to R280, which is at the surface of the protein, and then mutated the arginine 175 to histidine; after mutation, the mutp53^R175H^ was subjected to energy minimization using the OPLS3 force field (36) (Fig. S1*B*).

**Figure 1:**
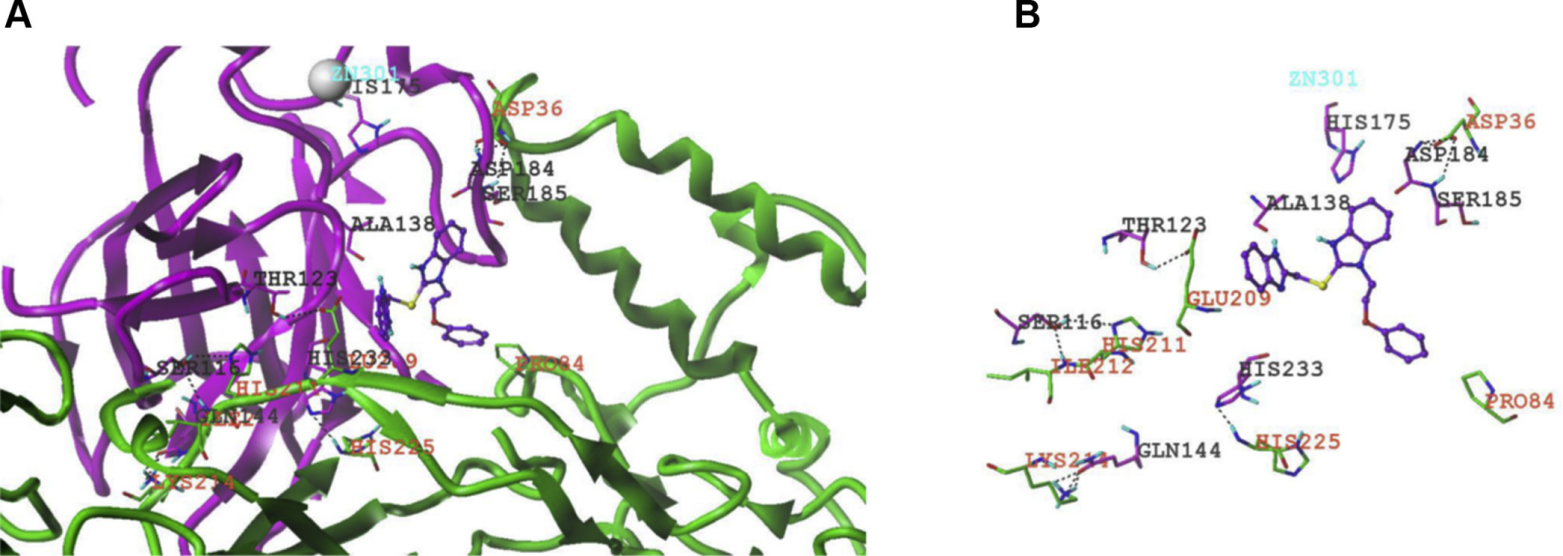
***In silico* analysis of 3D homology models, molecular docking site, and interacting pocket for DNAJA1–mutp53**^**R175H**^**protein complex.***A*, an interacting/druggable pocket of putative small molecule–binding site in DNAJA1–mutp53^R175H^ complex (*green structure*, DNAJA1; *magenta structure*, mutp53^R175H^). *B*, the interface between the interaction domains were shown along with the potential hydrogen bonds in between structures represented as *black dotted lines*. The small molecule ligand-binding site had also been shown in the interface between the two structures.

We then considered the validated DNAJA1 structure and the energy-minimized mutp53^R175H^ structure for a protein–protein docking experiment. As per the ZDOCK (37) protocols, we assigned DNAJA1, which has 397 amino acids, as the receptor and mutp53^R175H^ that has 192 amino acids to be the ligand. The designated ligand was rotated every 6° in the space of Euler angles around the receptor, and interacting energies were computed. This docking engine produced 2000 interacting poses. The poses were clustered, and 250 low-energetics poses of the two proteins were obtained. Analyzing these poses from the protein–protein docking runs, we found 10 low-energetics poses with similar binding modes and energies. We then generated the Connolly surface areas of the two interacting proteins and visualized any potential small molecule–binding sites or grooves in the interface between the two proteins. We observed that, of the 10 interacting poses, four of the poses or druggable interacting pockets showed putative small molecule–binding sites. Based on the interacting sites, we applied our in-house–generated artificial intelligence–based hotspots identification algorithm to identify the interacting hotspots between the two proteins. The pose that generated the highest number of hotspots (ΔΔG ≤ 2.0 kcal/mol) (38) was considered to be a critical docking site(s) or potential small molecule–binding site(s). It is intriguing that all four druggable interacting pockets for putative small molecule–binding sites were located at the only critical docking site of the DNAJA1 glycine/phenylalanine-rich region and at the non–DNA-binding surface of DNA-binding domain in mutp53^R175H^, as illustrated in Figure 1.

### Validation of the critical role of druggable binding sites and interacting pockets of the DNAJA1–mutP53^R175H^ complex in regulation of mutp53^R175H^ stabilization

**Figure 2:**
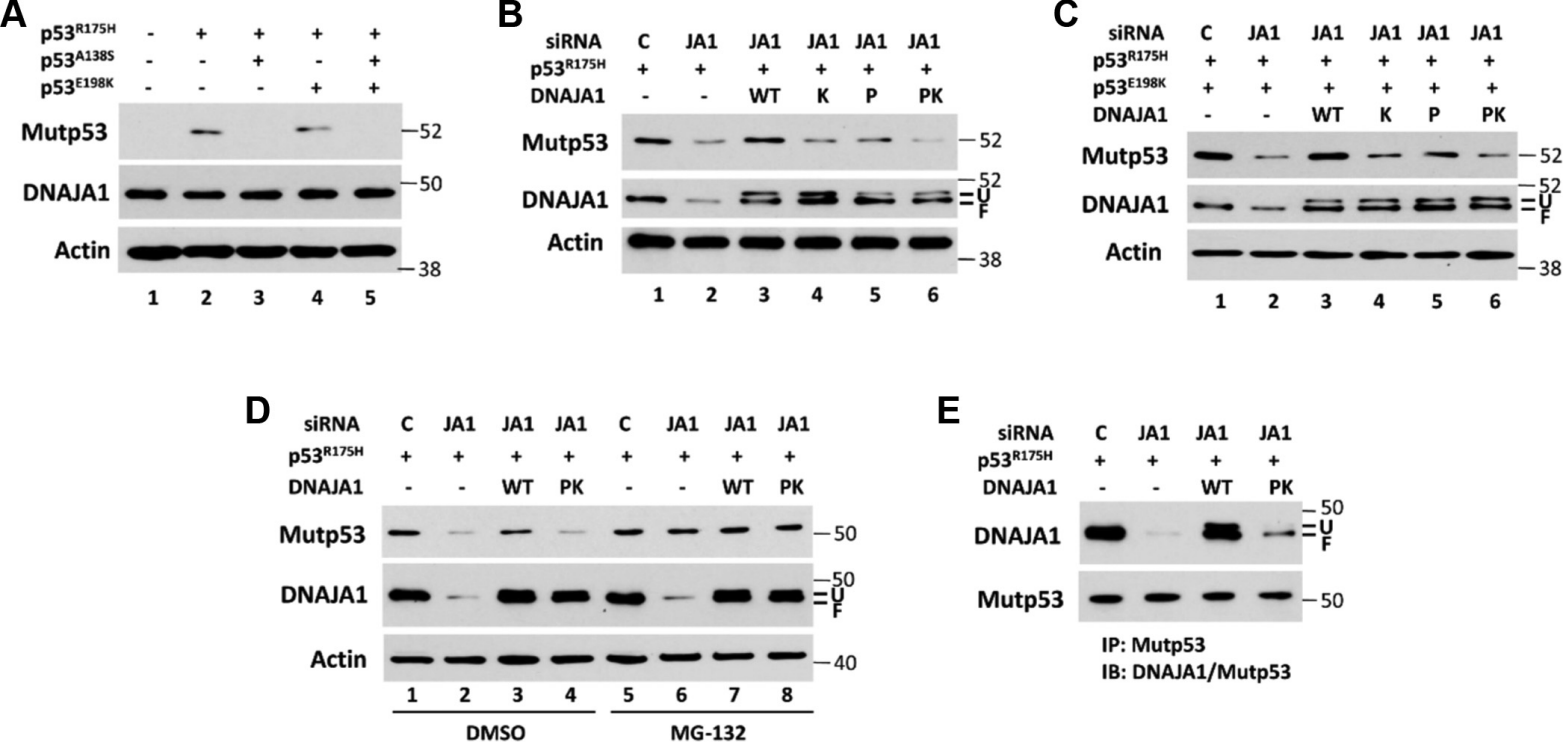
**Effects of different mutations of p53 and DNAJA1 on their interaction and mutp53 stability.***A*, human pancreatic cancer AsPC-1 cells (p53-null cells) were transfected with control plasmid (lane 1), mutp53^R175H^ plasmid (lane 2), mutp53R^175H/A138S^ plasmid (lane 3), mutp53^R175H/E198K^ plasmid (lane 4), or mutp53^R175H/A138S/E198K^ plasmid (lane 5) for 24 h, and the expression of mutp53 and DNAJA1 was determined by Western blotting (Mutp53 antibody: SC-99 from Santa Cruz Biotechnology). *B* and *C*, in AsPC-1 cells, the endogenous DNAJA1 expression was knocked down through siRNA, followed by transfection with mutp53^R175H^ or mutp53R^175H/E198K^ plasmid, together with wildtype (WT) DNAJA1 or different DNAJA1 mutation plasmids (K, K125Q mutation; P, P84S mutation; PK both P84S and K125Q mutation). Mutp53 and DNAJA1 protein levels were detected by Western blotting. *D*, AsPC-1 cells were treated as described for *B* and further treated with MG-132 (25 μM) or DMSO for another 4 h. Western blotting was performed to monitor mutp53 and DNAJA1 protein levels. *E*, co-immunoprecipitation to confirm the different interactions between p53^R175H^ and WT DNAJA1 or mutant DNAJA1 (PK). AsPc-1 cells were treated as described for *B* and immunoprecipitated by anti-mutp53 antibody, followed by Western blotting to detect DNAJA1 and mutp53 protein levels. F, farnesylated DNAJA1; U, unfarnesylated DNAJA1.

Since the interacting pockets in the DNAJA1–mutp53^R175H^ complex are in a druggable docking site of the DNAJA1 glycine/phenylalanine-rich region, we further analyzed whether the interacting pocket is critical for mutp53^R175H^ stability, particularly for the hotspots of Ala138 and Glu198 in mutp53^R175H^ and Pro84 and Lys125 in DNAJA1. Using a site-directed mutagenesis approach, we generated multiple mutant p53 constructs (p53^R175H/E198K^, p53^R175H/A138S^, and p53^R175H/A138S/E198K^) and DNAJA1 mutants (DNAJA1^K125Q^, DNAJA1^P84S^, and DNAJA1^P84S/K125Q^). AsPC-1 is a p53-null human pancreatic cancer cell line (39) into which we transfected different mutant p53 constructs. We found that AsPC-1 cells transfected with p53^R175H/A138S^ plasmid or p53^R175H/A138S/E198K^ plasmid led to loss of mutp53^R175H^ expression and transfection with p53^R75H/E198K^ plasmid resulted in decrease in mutp53^R175H^ expression, whereas DNAJA1 protein levels remained constant (Fig. 2*A*). To exclude the possibility that the loss of mutp53^R175H^ expression could be due to the A138S mutation that may prevent binding of this mutp53 antibody, we performed the same experiment with another mutp53 antibody, and the result was consistent (Fig. S2). Together, these results indicate that Glu198 and Ala138 are critical for mutp53^R175H^ stabilization. To test the effects of the interaction between mutp53^R175H^ and DNAJA1 on mutp53^R175H^ stability, we knocked down the endogenous DNAJA1 expression in AsPC-1 cells through DNAJA1 siRNA, followed by co-transfection of cells with p53^R175H^, p53^R175H/E198K^, wildtype (WT) DNAJA1, or mutant DNAJA1 plasmids (DNAJA1^K125Q^, DNAJA1^P84S^, and DNAJA1^P84S/K125Q^). As shown in Figure 2, *B–C*, siRNA knockdown of DNAJA1 resulted in a significant reduction of mutp53^R175H^ protein levels (lane 2 *versus* lane 1), which could be rescued by re-expression of WT DNAJA1 (lane 3 *versus* lane 2), and mutant DNAJA1 plasmids resulted in a significant decrease in mutp53^R175H^ protein levels, particularly in cells transfected with double mutant of P84S and K125Q (lanes 4, 5, and 6 *versus* lane 3). In view of the fact that DNAJA1 prevents E3 ligase CHIP-mediated ubiquitin proteasomal pathway to degrade mutp53 by competitively binding with mutp53 (24), after siRNA knockdown and plasmids transfection, AsPC-1 cells were further treated with MG-132, a proteasome inhibitor. As shown in Figure 2*D*, MG-132 dramatically increased mutp53 protein levels in AsPC-1 cells after knockdown of DNAJA1 or expression of mutant DNAJA1, whereas MG-132 had minimal effect on mutp53 levels in control AsPC-1 cells or AsPC-1 cells transfected with WT DNAJA1, indicating that DNAJA1 plays an important role in preventing proteasome-mediated mutp53 degradation and the predicted binding sites are crucial for DNAJA1 function to maintain mutp53 stability. To further determine whether these mutations truly impact DNAJA1–mutp53 interaction, we carried out an immunoprecipitation experiment and found that mutant DNAJA1 markedly reduced its binding to mutp53 (Fig. 2*E*).

**Figure 3:**
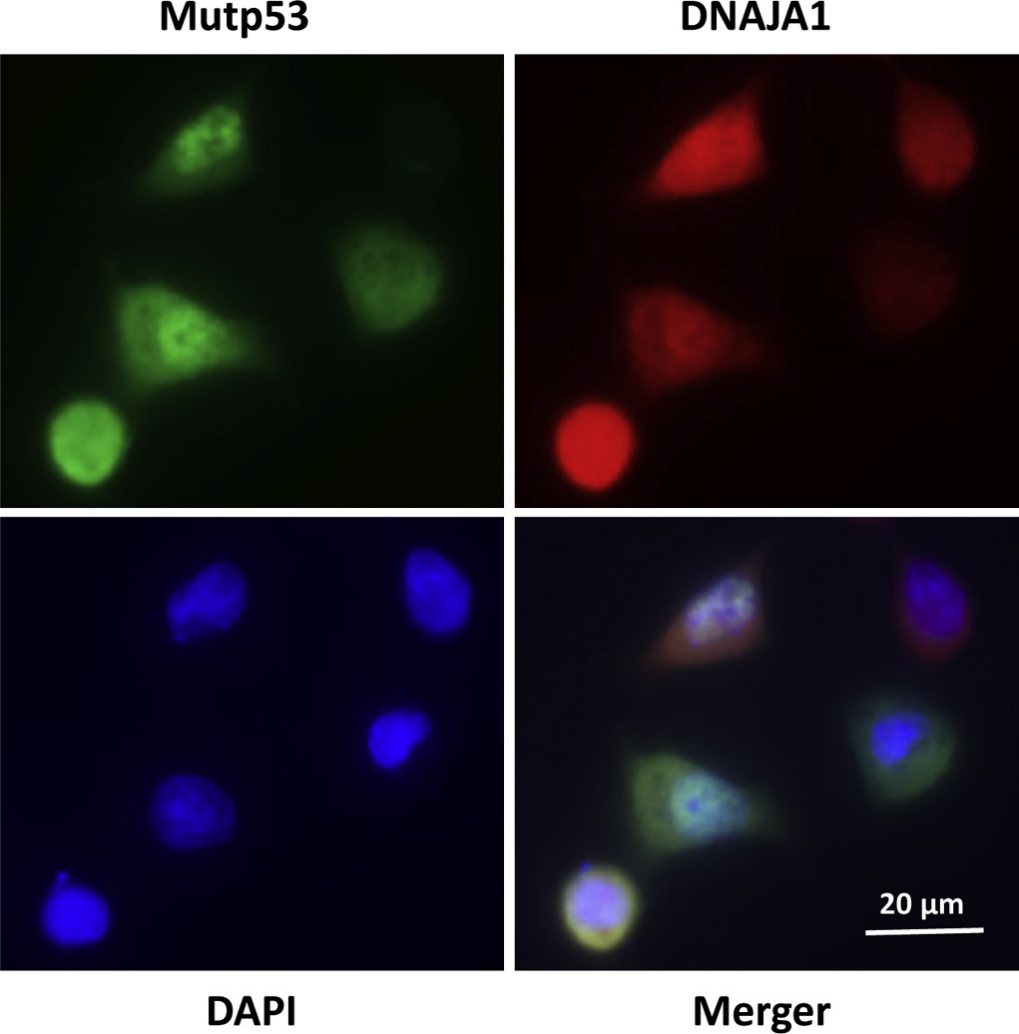
**Subcellular localization of p53**^**R175H**^**and DNAJA1 in AsPC-1 cells.** Cells were grown on chamber slides, transfected with p53^R175H^ plasmid, fixed with 4% paraformaldehyde in PBS, and immunostained with mutp53 (*green*), DNAJA1 (*red*), and DAPI (*blue*). (Scale bar is in the photo.)

To investigate the cellular localization of mutp53 and DNAJA1, immunofluorescence staining was performed in AsPC-1 cells after the cells were transfected with p53^R175H^ plasmid. As illustrated in Figure 3, DNAJA1 was located at both nuclear and cytoplasmic compartments as we previously reported in P03 cells (29). Of interest, unlike endogenous p53, which is mainly localized in nucleus, expression of p53^R175H^ was found in both nucleus and cytoplasm, although in many cells it was concentrated in nuclei. Collectively, these results demonstrate that the co-localization of DNAJA1 and p53^R175H^ and the identified docking sites/interacting pockets in the DNAJA1–mutP53^R175H^ complex are crucial for mutp53 stability. These results also indicate that the docking sites/interacting pockets are excellent candidate druggable sites.

### Small molecules identification through drug-like library screening against the DNAJA1–mutp53^R175H^ interacting pocket

**Figure 4:**
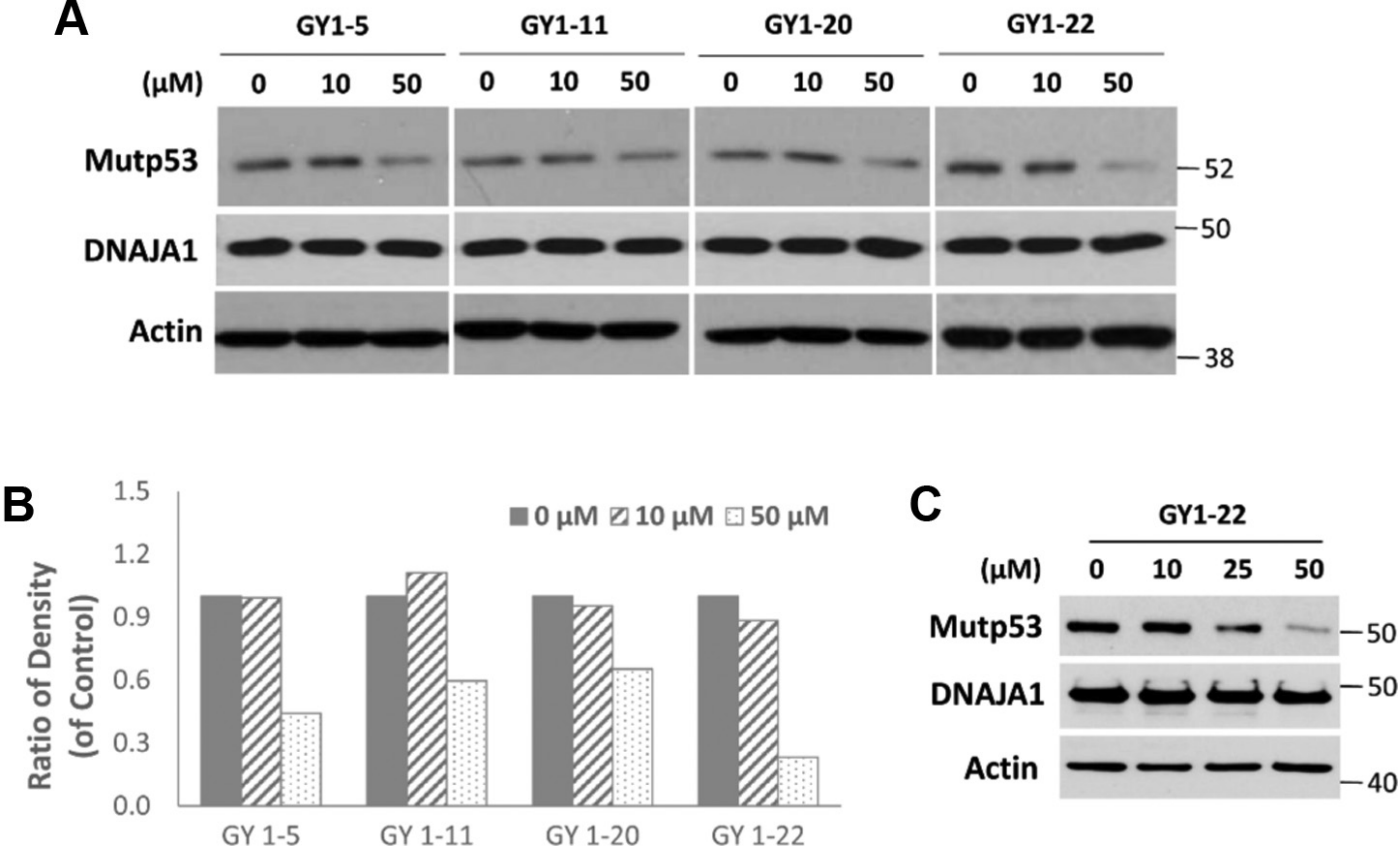
**Screening with small molecule drug-like library against DNAJA1–mutP53**^**R175H**^**interacting pocket.***A*, four of the 27 top hits showed the effect on reducing mutp53 protein level in mouse pancreatic cancer P03 cells. Cells were treated with different concentrations of the compounds for 24 h, and the protein levels of mutp53 and DNAJA1 were determined by Western blotting. *B*, the mutp53 band intensities were determined by densitometry and were normalized to loading control actin. *C*, human colon cancer LS123 cells (containing the p53^R175H^ mutation) were treated with different concentrations of GY1-22 for 24 h, and the protein levels of mutp53 and DNAJA1 were detected by Western blotting.

One of the key elements in any library screening is to ensure that the identified hit compounds are drug-like and chemically tractable. Often, hits identified by conventional high-throughput screening possess non–drug-like properties and are unsuitable for chemical modification. We created a curated small molecule database using multiple tiers of filters (such as Lipinski [40], Veber [41], and 239 PAINs [42]) from the ZINC database (43), which contains approximately 45 million purchasable compounds. This proprietary database has been previously used by us in many successful *in silico* screening studies (44, 45, 46, 47). Since we have identified and validated promising small molecule–binding pockets at a druggable docking site of DNAJA1 and at the interface of the DNAJA1–mutp53^R175H^ complex as mentioned above, we proceeded to screen a diverse library of 1 million compounds using the three-tiered Glide small molecule docking engine (48) from Schrödinger. The small molecule hit set obtained through Glide was cross-docked with Gold (49) and Surflex (50) docking engines, which are built upon orthogonal algorithms. We identified 27 of the top hit molecules (Glide score ≤6.0) interacting with the interface of the DNAJA1–mutp53^R175H^ interacting pocket. To validate the 27 hits, we used a murine pancreatic carcinoma cell model (P03 cells, containing a p53^R172H^ mutation, which equals human p53^R175H^ mutation). As illustrated in Figure 4, four hits showed a significant reduction of mutp53^R172H^ expression at a dose range of 10 to 50 μM, whereas DNAJA1 protein levels remained unchanged, and GY1-22 (chemical name: 2- {2-[(1H benzimidazol-2-ylmethyl)sulfanyl]-1H-benzimidazol-1-yl}ethyl phenyl ether) was the most promising hit. Not only in P03 cells but also in human colon cancer cell line LS123, which also contains p53^R175H^ mutation, GY1-22 reduced mutp53 protein expression (Fig. 4*C*).

### GY1-22 loses the ability to degrade mutp53^R175H^ after mutation of critical sites at the interface of the DNAJA1–mutp53^R175H^ complex

**Figure 5:**
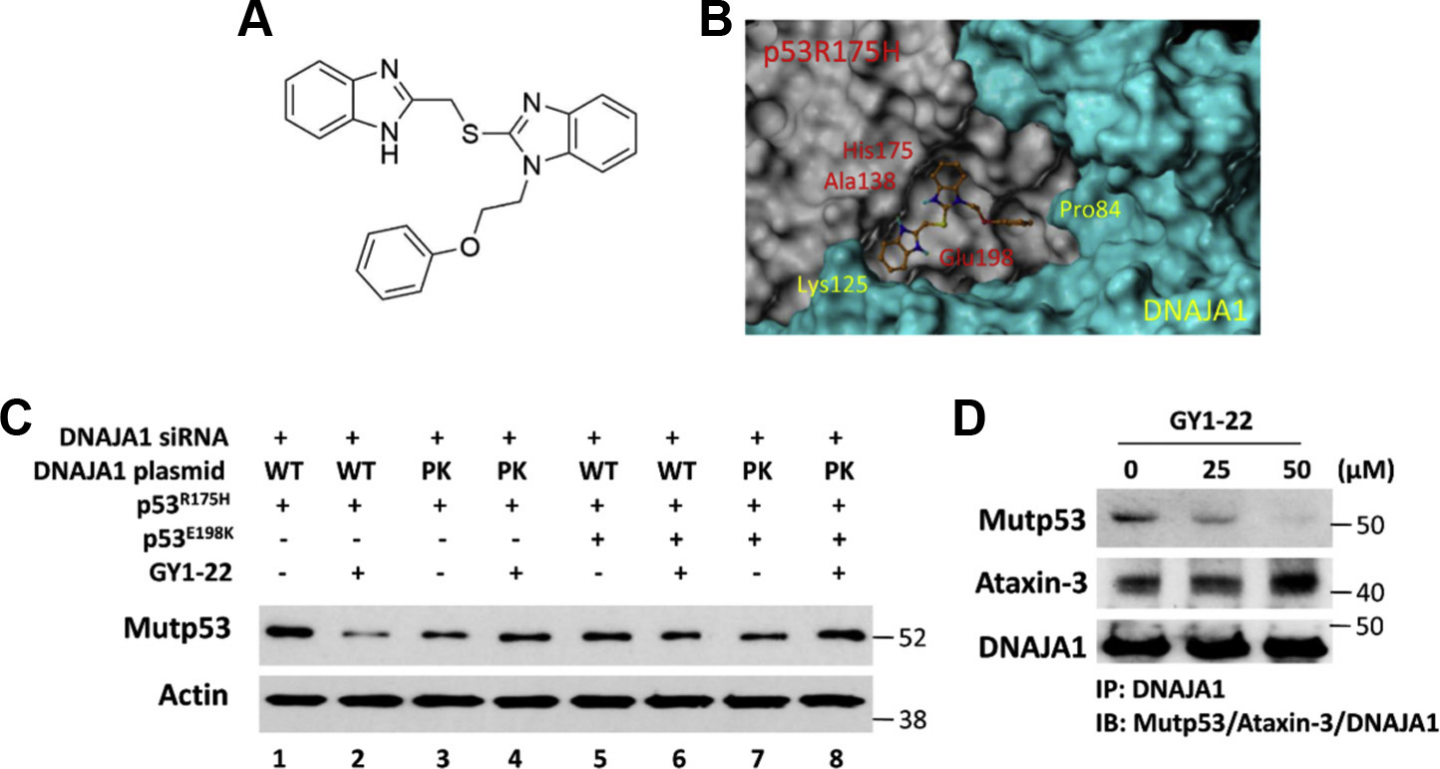
**Validation of the most promising hit GY1-22 on its ability to reduce mutp53 expression.***A*, chemical structure of GY1-22. *B*, GY1-22 docked pose on DNAJA1–mutp53^R175H^ complex. *C*, AsPC-1 cells were transfected with DNAJA1 siRNA to knock down endogenous DNAJA1 expression for 24 h, followed by co-transfection with mutp53^R175H^ or mutp53^R175H/E198K^ plasmids and wildtype (WT) DNAJA1 or DNAJA1 mutants (PK, both P84S and K125Q mutation) for another 24 h, then treated with DMSO or 25 μM GY1-22 for 24 h. *D*, P03 cells were treated with different concentrations of GY1-22 for 24 h and subjected to immunoprecipitation by anti-DNAJA1 antibody, followed by Western blotting to detect mutp53, ataxin-3, and DNAJA1 protein levels.

Studying the chemical structure of GY1-22 (Fig. 5*A*) by analyzing the GY1-22 docked pose, we observed that one of the benzimidazoles of the compound had two potential hydrogen bonds with an Ala138 backbone and a Glu198 side chain of mutp53^R175H^, whereas the phenyl ring of the compound showed a hydrophobic interaction with the Pro84 of the DNAJA1 druggable docking site in the glycine/phenylalanine-rich region (Fig. 5*B*). Using the site-directed mutagenesis approach and AsPC-1 cells, we further explored whether there were any effects on GY1-22–induced mutp53^R175H^ degradation by introducing the mutation at the docked binding site in the DNAJA1–mutp53^R175H^ complex. As shown in Figure 5*C*, after knockdown of DNAJA1 by siRNA, GY1-22 treatment (25 μM) resulted in a marked reduction of mutp53^R175H^ expression in AsPC-1 cells transfected with WT DNAJA1 and mutp53^R175H^ plasmids (lane 2 *versus* lane 1), whereas GY1-22 treatment lost the ability to reduce mutp53^R175H^ expression in AsPC-1 cells transfected with mutant DNAJA1 and mutp53^R175H^ plasmids (lane 4 *versus* lane 3). In addition, GY1-22 treatment did not show an effect on reducing mutp53^R175H^ expression in AsPC-1 cells transfected with the p53^R175H/E198K^ and WT DNAJA1 plasmids (lane 6 *versus* lane 5). Finally, AsPC-1 cells transfected with both p53^R175H/E198K^ and mutant DNAJA1 plasmids exhibited a decrease of mutp53^R175H^ expression (lane 7 *versus* lane 1), and subsequently treatment of GY1-22 did not display any effect on inducing mutp53^R175H^ degradation (lane 8 *versus* lanes 7). To investigate whether GY1-22 really disrupts the binding between DNAJA1 and mutp53, a co-immunoprecipitation experiment was performed. As shown in Figure 5*D*, compared with control (without GY1-22 treatment), GY1-22 significantly reduced the binding between DNAJA1 and mutp53 in a dose-dependent manner. Antaxin-3 is another protein that has been reported to bind to DNAJA1 (51); however, GY1-22 did not disrupt its binding to DNAJA1, indicating that GY1-22 specifically disrupts the binding between mutp53 and DNAJA1. Taken together, we identified and confirmed the specific binding site of compound GY1-22 on the DNAJA1–mutp53^R175H^ complex, which is important for moving forward in testing its biological effects.

### Biological effects of GY1-22 on mutp53-driven P03 pancreatic cancer cell growth *in vitro* and *in vivo*

**Figure 6:**
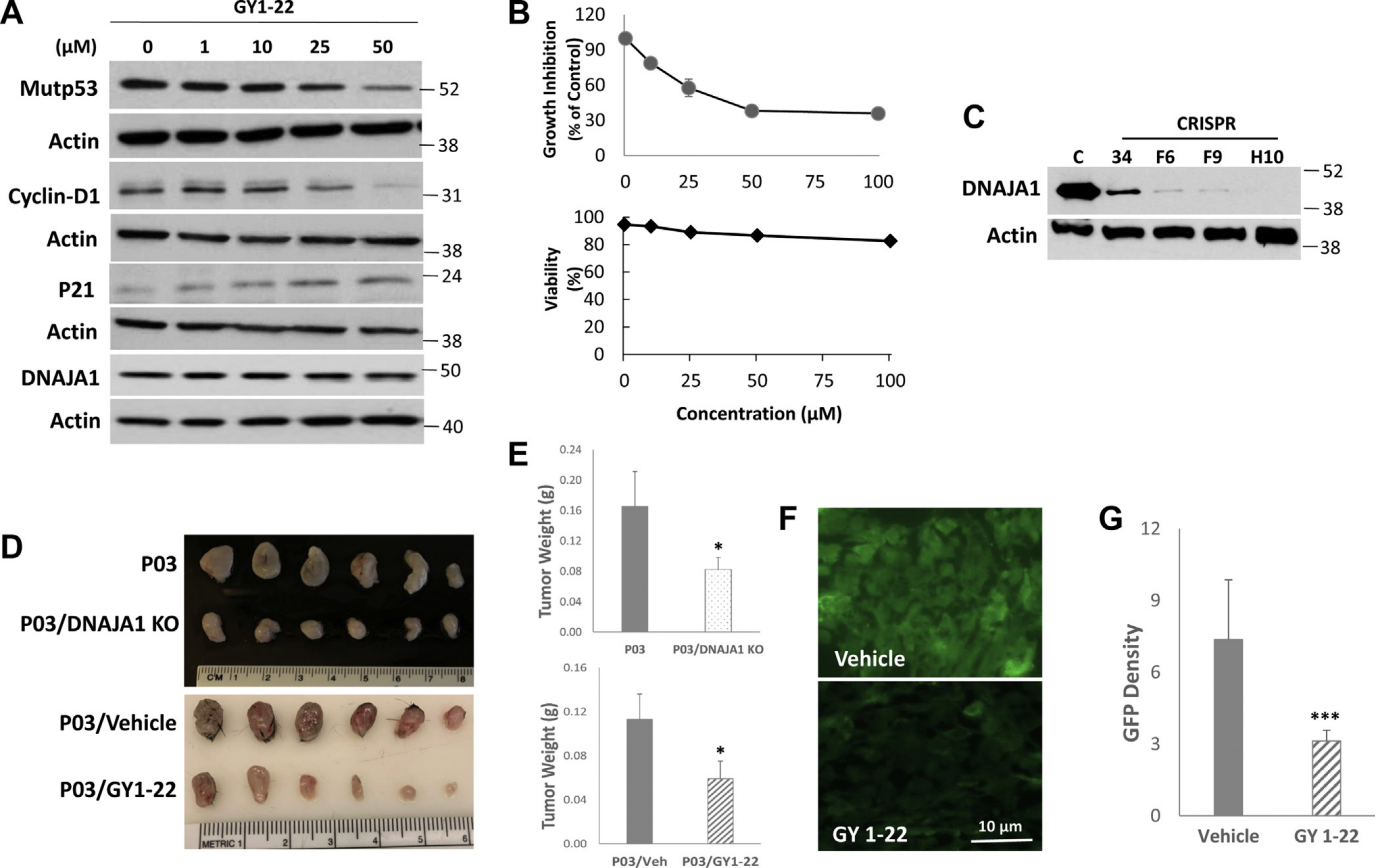
**Effects of GY1-22 on mutp53-driven P03 pancreatic cancer cell growth *in vitro* and *in vivo*.***A*, Western blotting assay for mutp53, cyclin D1, Waf1p21, and DNAJA1 after different doses of GY1-22 treatment for 24 h. *B*, *in vitro* growth inhibition (*upper histogram*) and cell viability (*lower histogram*) of P03 cells treated with GY1-22. *C*, knockout of DNAJA1 gene in P03 cells through CRISPR/Cas9 system. C, control P03 cells; 34, F6, F9, and H10 were different stable cell clones after transfection with DNAJA1 CRISPR/Cas9 KO plasmid, which encodes the Cas9 nuclease and DNAJA1-sepecific 20-nt guide RNA; DNAJA1 expression was completely shut down in clone H10. *D* and *E*, *in vivo* inhibition of P03 pancreatic cancer cell growth in transplanted C57BL/6J mice (n = 6 mice per dose, equal gender) either by shutdown of DNAJA1 gene expression (clone H10) or treatment with GY1-22 (1 mg/kg). *F* and *G*, immunofluorescent staining (scale bar in the photo) and density quantitation of mutp53 for *in vivo* P03 tumor treated without or with 1 mg/kg GY1-22 compound (∗*p* < 0.05; ∗∗∗*p* < 0.001).

Analysis of efficacy and cytotoxicity of GY1-22 was performed both *in vitro* and *in vivo*. In P03 cells (derived from mice carrying both wtp53 and mutp53 ^R172H^, equivalent to human mutp53^R175H^ [29]) treated with GY1-22 for 24 h *in vitro*, GY1-22 exhibited a dose-dependent effect on inhibition of mutp53 and cyclin D1 expression but induction of wtp53-activated Waf1p21 expression (Fig. 6*A*). In agreement with these findings, GY1-22 also showed a dose-dependent effect on inhibiting cell growth with IC_50_ 28 μM and low cytotoxicity (cell viability) (Fig. 6*B*). GY1-22 toxicity in rats (from the material safety data sheet) are LD_50_ = 1240 mg/kg, and the lowest observed adverse effect level/long-term toxic effect = 32.3 mg/kg. We performed a toxicity study by using 8- to 10-week-old C57BL/6J mice treated with 1, 3, and 10 mg/kg (daily i.p., three mice per dose) for 2 weeks. Dosage of 10 mg/kg (three times lower than the lowest observed adverse effect level) did not exhibit any toxicity grossly or histologically. To determine the role of DNAJA1 in mutp53^R175H^-driven carcinogenesis, we generated a DNAJA1 knockout (KO) P03 stable cell line through CRISPR/Cas9 system (Fig. 6*C* and S3), and either P03 or P03/DNAJA1 KO cells were transplanted into C57BL/6J mice (2 × 10^5^ cells per site, subcutaneous inoculation). DNAJA1 knockout alone exhibited a significant inhibition of tumor growth 2 weeks after transplantation (Fig. 6, *D*–*E*, upper panels). P03 subcutaneous implanted in C57BL/6J mice treated with GY1-22 at 1 mg/kg, i.p. injection (n = 6 mice), showed a significant inhibition of *in vivo* tumor growth (Fig. 6, *D*–*E*, lower panels), which was comparable with P03 DNAJA1 knockout line. Immunofluorescent staining of the tumors showed a significant decrease of mutp53, too (Fig. 6, *F–G*). Collectively, these data strongly demonstrate that the DNAJA1–mutp53 complex is a critical target for mutp53-driven cancer and GY1-22 is a novel small molecule inhibitor against the DNAJA1–mutp53^R175H^ complex with low toxicity.

## Discussion

Mutp53 can either abrogate tumor-suppressive function of wtp53 (loss of function) or acquire new oncogenic function (gain of function) to promote carcinogenesis. Therefore, targeting mutp53 for its degradation may serve as a promising therapeutic strategy for many cancers that harbor mutp53. We tested this strategy by studying whether mutp53 and its DNAJA1 chaperone complex are druggable targets for eradicating mutp53. Using an *in silico* approach, we built 3D homology models of human DNAJA1 and mutp53^R175H^ proteins and identified the interacting pocket(s) in the DNAJA1–mutp53^R175H^ complex and found only one critical druggable docking site in the DNAJA1 glycine/phenylalanine-rich region. We demonstrated that the interacting pocket in the DNAJA1–mutp53^R175H^ complex was crucial for stabilizing mutp53^R175H^ using a site-directed mutagenesis approach, indicating its potential as a druggable targeting site. We further screened a drug-like library to identify a promising hit (GY1-22) against the interacting pocket in the DNAJA1–mutp53^R175H^ complex. The GY1-22 compound displayed inhibitory activity against the DNAJA1–mutp53^R175H^ complex. In addition, treatment with GY1-22 significantly reduced mutp53 expression, enhanced Waf1p21 expression, suppressed cyclin D1 expression, and inhibited mutp53-driven pancreatic cancer growth both *in vitro* and *in vivo*, indicating that DNAJA1 is critical for chaperoning mutp53’s stability and oncogenic function and is a potential robust therapeutic target for developing the efficient small molecule inhibitors against oncogenic mutp53.

Besides loss of function and gain of function, mutp53 has a dominant negative effect through inactivation of wtp53 tumor-suppressive function, which occurs in many cancer models. For example, transgenic mice expressing mutp53 in p53^+/−^ background have a higher incidence of tumor formation and increased rate of metastasis compared with p53^+/−^ mice (12, 52). There are several underlying mechanisms that can explain the dominant negative effect: First, mutp53 has the ability to heterotetramarize with wtp53 and converts the wtp53 to an inactive, mutant conformation (53, 54). Second, it may be due to insufficient participation of mutp53 in transactivation of some p53 targets (54, 55). Third, mutp53 could bind to transcriptional cofactors that are required for wtp53 function (56). Since almost half of human cancers have *TP53* mutations and mutation of one allele of p53 can promote carcinogenesis due to the dominant negative effect, the best strategy to treat mutp53-containing cancer is to eliminate mutp53 and restore the tumor-suppressive function of wtp53. We found that, in P03 cells (derived from a mouse pancreatic carcinoma with mutp53^R172H^, equivalent to human mutp53^R175H^), treatment of GY1-22 significantly decreased the mutp53^R172H^ protein level and markedly enhanced Waf1p21 expression, a wtp53-activated gene, indicating that GY1-22 not only eliminates mutp53 but also restores wtp53 tumor-suppressive function by abrogating mutp53 dominant negative effect.

As we mentioned above, the interacting pocket in the DNAJA1–mutP53^R175H^ complex is crucial for mutp53 stability; particularly, the spots of Ala138 and Glu198 in mutp53^R175H^ and Pro84 and Lys125 in DNAJA1 play an important role in maintaining mutp53. Surprisingly, when we introduced another mutation (A138S) into mutP53^R175H^, it led to loss of expression of the new mutp53^R175H/A138S^ protein. The A138S mutation has been deposited in the latest IARC TP53 Database (R20, https://p53.iarc.fr/). It is located in *TP53* exon 5 and is due to the codon 412 G to T mutation. Unlike R175H mutation that loses wtp53 transactivation ability, the A138S missense mutation still keeps wtp53 transactivation function and can fully regulate many downstream target genes (57, 58). One function of the Hsp40/DNAJ family (DNAJA1 is a member of this protein family) is its involvement in refolding misfolded proteins to prevent them from being degraded by proteases. Given the fact that DNAJA1 inhibits the activities of CHIP on mutp53 by competitively binding with mutp53 (24), it is possible that A138S mutation may change the conformation of mutp53^R175H^, thereby making it impossible for DNAJA1 to bind with mutp53^R175H/A138S^, and thus no longer inhibiting the activity of CHIP on mutp53.

Previously, we have demonstrated that farnesylation of DNAJA1 at the C-terminal CAAX motif is critical for mutp53 stabilization and atorvastatin inhibits DNAJA1 farnesylation and promotes mutp53 degradation (29). In the current study, we further found that GY1-22 induces mutp53 degradation but through a different mechanism: binding pockets at a druggable docking site of the DNAJA1 glycine/phenylalanine-rich region and at the interface of the DNAJA1–mutp53^R175H^ complex. It would be very interesting to ask whether GY1-22 synergizes with atorvastatin on mutp53 degradation. Indeed, we found that, at 10 μM concentration, atorvastatin alone barely induced mutp53 degradation; however, cotreatment of atorvastatin with GY1-22 markedly reduced the mtp53 protein level. Furthermore, GY1-22 synergized with atorvastatin as low as 1 μM concentration (Fig. S4).

We identified GY1-22 by using one of the most frequent p53 mutations (R175H); however, DNAJA1 has been reported to interact with different mutp53 (24); whether GY1-22 has the same effect on other missense mutp53 remains to be elucidated. On the other hand, based on the findings presented here and in the literature to date, it is thus reasonable to suggest that more small molecules against a wide spectrum of mutp53 could be identified based on the druggable docking site in the DNAJA1 rather than the interacting pockets in the DNAJA1–mutp53^R175H^ complex.

## Experimental procedures

### Cell culture and drug treatment

The mouse pancreatic carcinoma cell P03 was derived from PDAC KPC ^R172H^ mouse, which has a R172H missense p53 mutation, equivalent to human R175H mutation. Cells were cultured in Dulbecco’s modified Eagle’s medium containing 10% fetal bovine serum (FBS) and 0.1% gentamicin. AsPC-1 is a p53-null human pancreatic carcinoma cell line and was cultured in RPMI-1640 medium with 10% FBS and 0.1% gentamicin. LS123 is a human colon cancer cell line (containing the p53^R175H^ mutation), grown in Eagle’s minimum essential medium with 10% FBS and 0.1% gentamicin. All cell lines were maintained at 37 °C with 5% CO_2_. MG-132 was from Sigma; other compounds (including GY1-22) were purchased from Chembridge Corp and reported to be >95% pure by the vendor. For drug treatment, different small molecules were dissolved in dimethyl sulfoxide (DMSO) and added into culture medium for 24 h; the control group was treated with DMSO only.

### Animal experiments

C57BL/6J mice were purchased from the Jackson laboratory. P03 cells or P03/DNAJA1 KO cells (2 × 10^5^ cells per 100 μl plain medium per site) were injected subcutaneously into both sides of flank. To test the GY1-22 effect, only P03 cells transplanted into mice were used. On the next day, GY1-22 was administered to mice by i.p. injection daily in the dose of 1 mg/kg body weight; control group animals were injected with DMSO. Tumor development was monitored daily, and mice were sacrificed at 17 days post P03 cell transplantation. Tumors were dissected and measured for size (length and width) and weight. Mice were housed under pathogen-free conditions with free access to water and food in the animal facility at the Center for Comparative Medicine at Northwestern University. All research involving animals have been reviewed and approved by the Northwestern University Animal Care and Use Committee and were conducted in compliance with Northwestern University IACUC guidelines.

### Tissue preparation and immunofluorescence

Mice were euthanized with CO_2_ and the tumors were collected, fixed in 10% formalin for 24 h, routinely processed, and embedded in paraffin. Five-micrometer serial paraffin sections were obtained on poly-l-lysine–coated slides. For tissue immunofluorescence staining, paraffin sections were rehydrated and antigens were retrieved using citrate buffer in a microwave. The horse serum was used to block nonspecific protein interactions. Slides were then incubated with primary antibody (p53 (CM5) antibody, Vector Labs) at 4 °C overnight, followed by incubation with Alexa Fluor 488–conjugated secondary antibody (Thermo Fisher Scientific) for 1 h at room temperature. After the final wash with PBS, the slides were mounted and visualized with a fluorescence microscope.

### Western blot

After treatment, culture medium was removed and cells were washed with PBS, scraped, and lysed on ice for 30 min by using lysis buffer containing 1% PMSF, 1% protease inhibitor cocktail, and 1% phosphatase inhibitor cocktails 2 and 3 (Millipore-Sigma). The extracts were centrifuged at 13,000 rpm for 10 min at 4 °C. The supernatant was collected. Protein concentrations were determined by the BCA assay (Thermo Fisher Scientific). Twenty micrograms of protein was loaded on SDS-PAGE and transferred to the PVDF Membrane (Bio-Rad). The membrane was blocked with 5% non-fat dry milk in 1X TBST for 1 h at room temperature and followed by incubation with the primary antibody solution overnight at 4 °C. The membrane was then washed with TBST and incubated with HRP-linked secondary antibodies for 1 h at room temperature. The antibody-antigen complexes were detected by using the LumiGLO chemiluminescent substrate (Cell Signaling Technology) according to the manufacturer’s directions, and the emitted light was captured on X-ray film. The following antibodies were used: p53 (SC-99), p53 (SC-126), ataxin-3 (sc-398114), cyclin D1(SC-718), and p21 (SC-6246) from Santa Cruz Biotechnology; DNAJA1 (MS-225-P1) from Thermo Fisher Scientific; and β-actin from Sigma-Aldrich.

### Co-immunoprecipitation

Agarose-conjugated mutp53 antibody was purchased from Santa Cruz Biotechnology (SC-99 AC); agarose-conjugated DNAJA1 antibody was prepared by incubation of DNAJA1 antibody with protein A/G agarose beads on rotator for 4 h at 4 °C. Cell lysates were incubated with agarose-conjugated antibody overnight on rotator at 4 °C. Samples were centrifuged at 2000*g* for 3 min at 4 °C, and the pelleted beads were washed 4 times with cold wash buffer supplemented with protease inhibitors. Finally, 25 μl loading buffer was added to each sample, followed by SDS-PAGE and Western blot.

### Cellular immunofluorescence

AsPC-1 cells were plated into a 2-well chamber slide (Nunc Lab-Tek II) at the density of 8 × 10^4^ cells/ml. One day later, cells were transfected with p53^R175H^ plasmid for 24 h. Cells were washed with PBS, fixed by 4% paraformaldehyde for 15 min, followed by permeabilization with 0.1% Triton X-100 in PBS for 10 min at room temperature, and blocked by 3% BSA in PBS containing 0.1% Triton X-100 for 1 h at room temperature. Cells were then incubated overnight with anti-p53 (SC-99) antibody and anti-DNAJA1 antibody, followed by incubation with Alexa 568– and Alexa 488–conjugated secondary antibodies (Thermo Fisher Scientific) for 1 h at room temperature. After mounting with proLong Gold Antifade Mountant with DAPI (Invitrogen), immunofluorescence images were captured by fluorescence microscopy.

### RNA interference

Target-specific siRNA duplex targeting human DNAJA1 mRNA (SASI_Hs01_00031818) and non-targeting control siRNA (SIC001) were purchased from Millipore-Sigma. The transfection of siRNA into cells was performed by using Lipofectamine RNAiMAX (Thermo Fisher Scientific) according to the manufacturer’s instructions; cells were transfected for 24 h.

### Plasmids and site-directed mutagenesis

pCMV-Neo-Bam p53 R175H was a gift from Bert Vogelstein (Addgene plasmid # 16436), and pcDNA5/FRT/TO HIS DNAJA1 was a gift from Harm Kampinga (Addgene plasmid # 19545). The A138S and E198K mutations of p53^R175H^ and P84S and K125Q mutations of DNAJA1 were produced by QuikChange Multi Site-directed Mutagenesis kit (Agilent Technologies) to mutate codon 138 GCC (Ala) to TCC (Ser) (mutagenic primer: atgttttgccaactgTccaagacctgccctgtg), codon 198 GAA (Glu) to AAA (Lys) (mutagenic primer: gcatcttatccgagtgAaaggaaatttgcgtgtgg), codon 84 CCC (Pro) to TCC (Ser) (mutagenic primer: ggcggttttggctccTccatggacatctttg), and codon 125 AAA (Lys) to CAA (Gln) (mutagenic primer: atggtgcaacaagaCaactggctctgc), respectively. All mutations were confirmed by DNA sequencing.

### DNA transfection

The transfection of plasmids into AsPC-1 cells was performed by using Lipofectamine 3000 (Thermo Fisher Scientific) according to the manufacturer’s instructions. For some experiments, cells were first transfected with DNAJA1 siRNA to knock down endogenous DNAJA1 expression for 24 h, followed by co-transfection of different p53 mutants and WT DNAJA1 or DNAJA1 mutants for another 24 h.

### DNAJA1 gene knockout by CRISPR/Cas9 and stable cell line generation

DNAJA1 CRISPR guide RNA plasmid, which encodes the Cas9 nuclease and mouse DNAJA1-sepecific 20-nt guide RNA (TTTACCTTGTAAAAACAGCA, targeting DNAJA1 exon 6), were from GenScript; P03 cells were transfected with this plasmid by using Lipofectamine 3000. At 4 days after transfection, puromycin (3 μg/ml) was added into the culture medium to select stable cell lines. After the first round of selection, a pool of cells with dramatically reduced DNAJA1 expression was established and a monoclonal cell line was further isolated from the pool by limiting dilution. Each clonal cell line was detected by Western blot for the expression of DNAJA1 protein and for positive clones; the exact genomic mutation was determined by sequencing.

### Statistical analysis

Statistical analyses were carried out using Prism 6 (GraphPad software). All experiments were repeated at least three times; for Western blot and immunofluorescence image, a representative result was presented. The values were expressed as means ± SE. A probability value *p* < 0.05 was considered to be statistically significant. Normally distributed data were analyzed by using two-tailed Student’s *t* test.

## Data availability

All the data supporting our conclusions are presented in this article.

## Conflict of interest

The authors declare that they have no conflicts of interest with the contents of this article.

## References

[1] Vogelstein B., Lane D., Levine A.J. (2000). Surfing the p53 network. Nature.

[2] Ciriello G., Miller M.L., Aksoy B.A., Senbabaoglu Y., Schultz N., Sander C. (2013). Emerging landscape of oncogenic signatures across human cancers. Nat. Genet..

[3] Wade M., Li Y.C., Wahl G.M. (2013). MDM2, MDMX and p53 in oncogenesis and cancer therapy. Nat. Rev. Cancer.

[4] Kastenhuber E.R., Lowe S.W. (2017). Putting p53 in context. Cell.

[5] Baugh E.H., Ke H., Levine A.J., Bonneau R.A., Chan C.S. (2018). Why are there hotspot mutations in the TP53 gene in human cancers?. Cell Death Differ..

[6] Cho Y., Gorina S., Jeffrey P.D., Pavletich N.P. (1994). Crystal structure of a p53 tumor suppressor-DNA complex: understanding tumorigenic mutations. Science.

[7] Rolley N., Butcher S., Milner J. (1995). Specific DNA binding by different classes of human p53 mutants. Oncogene.

[8] Rivlin N., Brosh R., Oren M., Rotter V. (2011). Mutations in the p53 tumor suppressor gene: important milestones at the various steps of tumorigenesis. Genes Cancer.

[9] Muller P.A., Vousden K.H. (2014). Mutant p53 in cancer: new functions and therapeutic opportunities. Cancer Cell.

[10] Boettcher S., Miller P.G., Sharma R., McConkey M., Leventhal M., Krivtsov A.V., Giacomelli A.O., Wong W., Kim J., Chao S., Kurppa K.J., Yang X., Milenkowic K., Piccioni F., Root D.E. (2019). A dominant-negative effect drives selection of TP53 missense mutations in myeloid malignancies. Science.

[11] Lee M.K., Teoh W.W., Phang B.H., Tong W.M., Wang Z.Q., Sabapathy K. (2012). Cell-type, dose, and mutation-type specificity dictate mutant p53 functions *in vivo*. Cancer Cell.

[12] Liu G., McDonnell T.J., Montes de Oca Luna R., Kapoor M., Mims B., El-Naggar A.K., Lozano G. (2000). High metastatic potential in mice inheriting a targeted p53 missense mutation. Proc. Natl. Acad. Sci. U. S. A..

[13] Olive K.P., Tuveson D.A., Ruhe Z.C., Yin B., Willis N.A., Bronson R.T., Crowley D., Jacks T. (2004). Mutant p53 gain of function in two mouse models of Li-Fraumeni syndrome. Cell.

[14] Brosh R., Rotter V. (2009). When mutants gain new powers: news from the mutant p53 field. Nat. Rev. Cancer.

[15] Barta J.A., McMahon S.B. (2019). Lung-enriched mutations in the p53 tumor suppressor: a paradigm for tissue-specific gain of oncogenic function. Mol. Cancer Res..

[16] Datta A., Ghatak D., Das S., Banerjee T., Paul A., Butti R., Gorain M., Ghuwalewala S., Roychowdhury A., Alam S.K., Das P., Chatterjee R., Dasgupta M., Panda C.K., Kundu G.C. (2017). p53 gain-of-function mutations increase Cdc7-dependent replication initiation. EMBO Rep..

[17] Kim M.P., Lozano G. (2018). Mutant p53 partners in crime. Cell Death Differ..

[18] Lavin M.F., Gueven N. (2006). The complexity of p53 stabilization and activation. Cell Death Differ..

[19] Kubbutat M.H., Jones S.N., Vousden K.H. (1997). Regulation of p53 stability by Mdm2. Nature.

[20] Michael D., Oren M. (2003). The p53-Mdm2 module and the ubiquitin system. Semin. Cancer Biol..

[21] Jeng W., Lee S., Sung N., Lee J., Tsai F.T. (2015). Molecular chaperones: guardians of the proteome in normal and disease states. F1000Res.

[22] Gumeni S., Evangelakou Z., Gorgoulis V.G., Trougakos I.P. (2017). Proteome stability as a key factor of genome integrity. Int. J. Mol. Sci..

[23] Kim Y.E., Hipp M.S., Bracher A., Hayer-Hartl M., Hartl F.U. (2013). Molecular chaperone functions in protein folding and proteostasis. Annu. Rev. Biochem..

[24] Parrales A., Ranjan A., Iyer S.V., Padhye S., Weir S.J., Roy A., Iwakuma T. (2016). DNAJA1 controls the fate of misfolded mutant p53 through the mevalonate pathway. Nat. Cell Biol..

[25] Tracz-Gaszewska Z., Klimczak M., Biecek P., Herok M., Kosinski M., Olszewski M.B., Czerwinska P., Wiech M., Wiznerowicz M., Zylicz A., Zylicz M., Wawrzynow B. (2017). Molecular chaperones in the acquisition of cancer cell chemoresistance with mutated TP53 and MDM2 up-regulation. Oncotarget.

[26] Wawrzynow B., Zylicz A., Zylicz M. (2018). Chaperoning the guardian of the genome. The two-faced role of molecular chaperones in p53 tumor suppressor action. Biochim. Biophys. Acta Rev. Cancer.

[27] Terada K., Oike Y. (2010). Multiple molecules of Hsc70 and a dimer of DjA1 independently bind to an unfolded protein. J. Biol. Chem..

[28] Qiu X.B., Shao Y.M., Miao S., Wang L. (2006). The diversity of the DnaJ/Hsp40 family, the crucial partners for Hsp70 chaperones. Cell Mol. Life Sci..

[29] Xu D., Tong X., Sun L., Li H., Jones R.D., Liao J., Yang G.Y. (2019). Inhibition of mutant Kras and p53-driven pancreatic carcinogenesis by atorvastatin: mainly via targeting of the farnesylated DNAJA1 in chaperoning mutant p53. Mol. Carcinog..

[30] Sterrenberg J.N., Blatch G.L., Edkins A.L. (2011). Human DNAJ in cancer and stem cells. Cancer Lett..

[31] Wright L.P., Philips M.R. (2006). Thematic review series: lipid posttranslational modifications. CAAX modification and membrane targeting of Ras. J. Lipid Res..

[32] Caplan A.J., Tsai J., Casey P.J., Douglas M.G. (1992). Farnesylation of YDJ1p is required for function at elevated growth temperatures in *Saccharomyces cerevisiae*. J. Biol. Chem..

[33] Kanazawa M., Terada K., Kato S., Mori M. (1997). HSDJ, a human homolog of DnaJ, is farnesylated and is involved in protein import into mitochondria. J. Biochem..

[34] Farid R., Day T., Friesner R.A., Pearlstein R.A. (2006). New insights about HERG blockade obtained from protein modeling, potential energy mapping, and docking studies. Bioorg. Med. Chem..

[35] Chen V.B., Arendall W.B., Headd J.J., Keedy D.A., Immormino R.M., Kapral G.J., Murray L.W., Richardson J.S., Richardson D.C. (2010). MolProbity: all-atom structure validation for macromolecular crystallography. Acta Crystallogr. D Biol. Crystallogr..

[36] Harder E., Damm W., Maple J., Wu C., Reboul M., Xiang J.Y., Wang L., Lupyan D., Dahlgren M.K., Knight J.L., Kaus J.W., Cerutti D.S., Krilov G., Jorgensen W.L., Abel R. (2016). OPLS3: a force field providing broad coverage of drug-like small molecules and proteins. J. Chem. Theor. Comput..

[37] Wiehe K., Pierce B., Mintseris J., Tong W.W., Anderson R., Chen R., Weng Z. (2005). ZDOCK and RDOCK performance in CAPRI rounds 3, 4, and 5. Proteins.

[38] Moreira I.S., Fernandes P.A., Ramos M.J. (2007). Hot spots--a review of the protein-protein interface determinant amino-acid residues. Proteins.

[39] Rodicker F., Putzer B.M. (2003). p73 is effective in p53-null pancreatic cancer cells resistant to wild-type TP53 gene replacement. Cancer Res..

[40] Lipinski C.A. (2004). Lead- and drug-like compounds: the rule-of-five revolution. Drug Discov. Today Technol..

[41] Wan-Mamat W.M., Isa N.A., Wahab H.A., Wan-Mamat W.M. (2009). Drug-like and non drug-like pattern classification based on simple topology descriptor using hybrid neural network. Conf. Proc. IEEE Eng. Med. Biol. Soc..

[42] Baell J.B., Holloway G.A. (2010). New substructure filters for removal of pan assay interference compounds (PAINS) from screening libraries and for their exclusion in bioassays. J. Med. Chem..

[43] Sterling T., Irwin J.J. (2015). ZINC 15--ligand discovery for everyone. J. Chem. Inf. Model..

[44] Mishra R.K., Wei C., Hresko R.C., Bajpai R., Heitmeier M., Matulis S.M., Nooka A.K., Rosen S.T., Hruz P.W., Schiltz G.E., Shanmugam M. (2015). In silico modeling-based identification of glucose transporter 4 (GLUT4)-selective inhibitors for cancer therapy. J. Biol. Chem..

[45] Mishra R.K., Shum A.K., Platanias L.C., Miller R.J., Schiltz G.E. (2016). Discovery and characterization of novel small-molecule CXCR4 receptor agonists and antagonists. Sci. Rep..

[46] Mishra R.K., Singh J. (2015). A structure guided QSAR: a rapid and accurate technique to predict IC50: a case study. Curr. Comput. Aided Drug Des..

[47] Villa S.R., Mishra R.K., Zapater J.L., Priyadarshini M., Gilchrist A., Mancebo H., Schiltz G.E., Layden B.T. (2017). Homology modeling of FFA2 identifies novel agonists that potentiate insulin secretion. J. Investig. Med..

[48] Sherman W., Day T., Jacobson M.P., Friesner R.A., Farid R. (2006). Novel procedure for modeling ligand/receptor induced fit effects. J. Med. Chem..

[49] Verdonk M.L., Chessari G., Cole J.C., Hartshorn M.J., Murray C.W., Nissink J.W., Taylor R.D., Taylor R. (2005). Modeling water molecules in protein-ligand docking using GOLD. J. Med. Chem..

[50] Jain A.N. (2003). Surflex: fully automatic flexible molecular docking using a molecular similarity-based search engine. J. Med. Chem..

[51] Jana N.R., Nukina N. (2004). Misfolding promotes the ubiquitination of polyglutamine-expanded ataxin-3, the defective gene product in SCA3/MJD. Neurotox. Res..

[52] Harvey M., Vogel H., Morris D., Bradley A., Bernstein A., Donehower L.A. (1995). A mutant p53 transgene accelerates tumour development in heterozygous but not nullizygous p53-deficient mice. Nat. Genet..

[53] Milner J., Medcalf E.A. (1991). Cotranslation of activated mutant p53 with wild type drives the wild-type p53 protein into the mutant conformation. Cell.

[54] Chene P. (1998). *In vitro* analysis of the dominant negative effect of p53 mutants. J. Mol. Biol..

[55] Nicholls C.D., McLure K.G., Shields M.A., Lee P.W. (2002). Biogenesis of p53 involves cotranslational dimerization of monomers and posttranslational dimerization of dimers. Implications on the dominant negative effect. J. Biol. Chem..

[56] Joers A., Kristjuhan A., Kadaja L., Maimets T. (1998). Tumour associated mutants of p53 can inhibit transcriptional activity of p53 without heterooligomerization. Oncogene.

[57] Kato S., Han S.Y., Liu W., Otsuka K., Shibata H., Kanamaru R., Ishioka C. (2003). Understanding the function-structure and function-mutation relationships of p53 tumor suppressor protein by high-resolution missense mutation analysis. Proc. Natl. Acad. Sci. U. S. A..

[58] Kakudo Y., Shibata H., Otsuka K., Kato S., Ishioka C. (2005). Lack of correlation between p53-dependent transcriptional activity and the ability to induce apoptosis among 179 mutant p53s. Cancer Res..

