# Supplementary material for: Identification of a druggable protein–protein interaction site between mutant p53 and its stabilizing chaperone DNAJA1
Source: J Biol Chem. 2020 Nov 21;296:100098. doi: 10.1074/jbc.RA120.014749 (PMC7948449; doi:10.1074/jbc.RA120.014749)
Supplement: Supplementary file 1 — Figures S1 to S4 [file mmc1.docx]

**Identification of a druggable protein-protein interaction site between mutant p53 and its stabilizing chaperone DNAJA1**

Xin Tong^1,2^, Dandan Xu^1^, Rama K Mishra^3,4,5^, Ryan D Jones^1^, Leyu Sun^1^, Gary E Schiltz^2,3,4^, Jie Liao^1^, and Guang-Yu Yang^1,2*^

**Supporting Information**


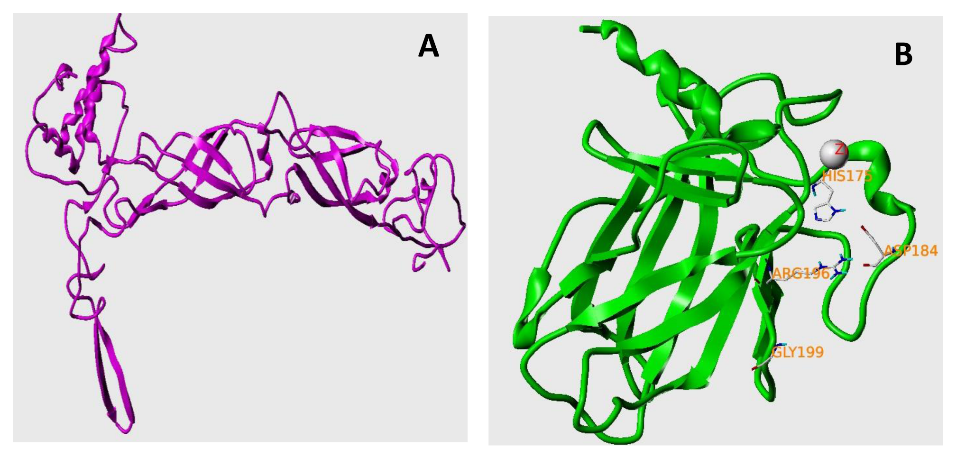


**Figure S1. Homology 3-D models of DNAJA1 protein (A) and mutp53^R175H^ protein (B).**


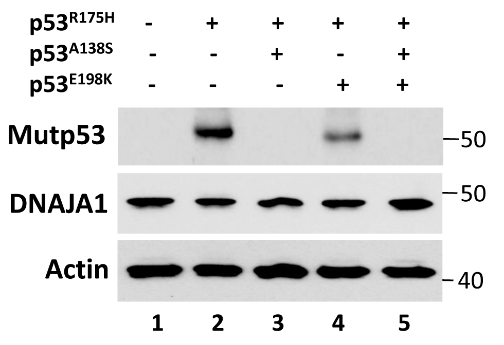


**Figure S2. Effects of different mutations of p53 on mutp53 protein stability.** Human pancreatic cancer AsPC-1 cells (p53-null cells) were transfected with control plasmid (lane 1), mutp53^R175H^ plasmid (lane 2), mutp53R^175H/A138S^ plasmid (lane 3), mutp53^R175H/E198K^ plasmid (lane 4) or mutp53^R175H/A138S/E198K^ plasmid (lane 5) for 24 h, and mutp53 expression was determined through Western blotting by using a different mut53 antibody (SC-126 from Santa Cruz Biotechnology).


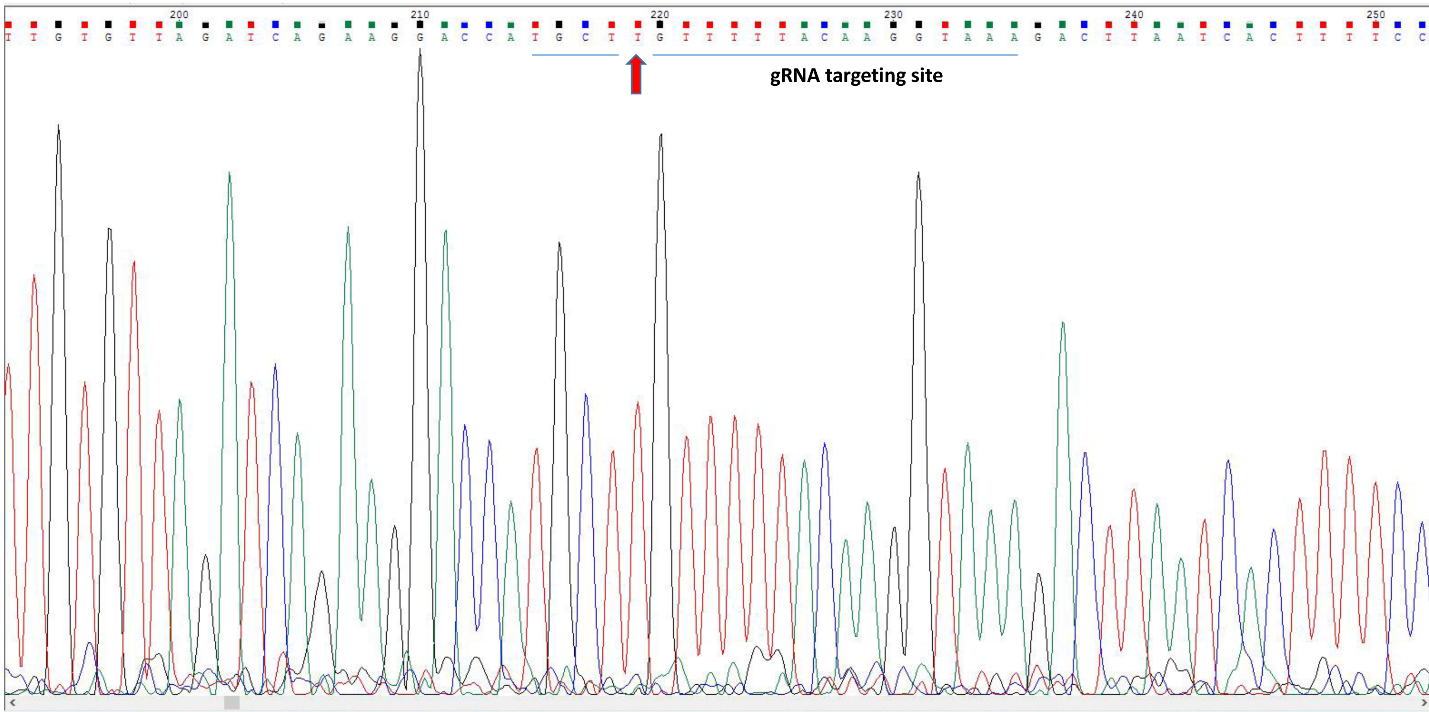


**Figure S3. Genomic DNA sequening result of the CRISPR-edited DNAJA1 gene (from clone H10).** The gRNA (TTTACCTTGTAAAAACAGCA) targets the end of mouse DNAJA1 Exon 6. After CRISPR gene editing, a “T” was inserted into the gRNA targeting site, resulted in frameshift and a premature stop codon “TGA” in the middle of Exon 7.


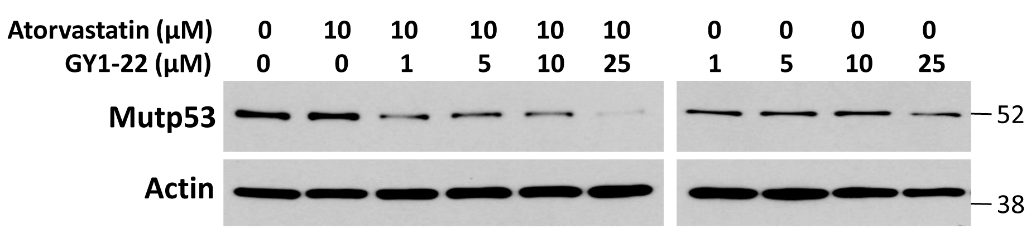


**Figure S4. Synergistic effect of GY1-22 and atorvastatin on mutp53 degradation.** P03 cells were treated with atorvastatin, GY1-22 alone or combination of them, mutp53 levels were detected by Western blotting at 24 h after drug treatment.
